# Seroprevalence of Hepatitis C Virus Among Blood Donors in a Tribal-Preponderant Region of India

**DOI:** 10.7759/cureus.62934

**Published:** 2024-06-22

**Authors:** Sushma Kumari, Anupa Prasad, Usha Saroj, Pramod Kumar, Saket Verma, Kumari Asha Kiran, Divakar Kumar

**Affiliations:** 1 Transfusion Medicine and Blood Bank, Rajendra Institute of Medical Sciences, Ranchi, IND; 2 Biochemistry, Rajendra Institute of Medical Sciences, Ranchi, IND; 3 Blood Bank, Rajendra Institute of Medical Sciences, Ranchi, IND; 4 Biochemistry, Hi-Tech Medical College and Hospital, Bhubaneswar, IND; 5 Biochemistry (Trauma Centre), Rajendra Institute of Medical Sciences, Ranchi, IND; 6 Preventive Medicine, Rajendra Institute of Medical Sciences, Ranchi, IND; 7 Internal Medicine, Rajendra Institute of Medical Sciences, Ranchi, IND

**Keywords:** tribal region in india, hepatitis c virus (hcv), blood donors, prevalence, trend

## Abstract

Introduction: Hepatitis C is a global health burden with significant morbidity and mortality. It primarily affects the liver and causes acute hepatitis, chronic hepatitis, liver cirrhosis, and hepatocellular carcinoma. Common modes of transmission of hepatitis C virus (HCV) infection are blood transfusion, needlestick injury, and mother-fetus transmission, among which transmission, blood transfusion is one of the most important causes. Blood transfusion is one of the pillars in the management of patients that saves lives and improves morbidity. Blood donation in India is done by voluntary and replacement blood donors of both sexes. The aim of this study is to determine the seroprevalence of HCV among blood donors in the Jharkhand state, a tribal-preponderant region of India, and to see the trend over the years.

Material and methods: This is a nine-year retrospective observational study from 2015 to 2023 that screened for anti-HCV antibodies (third-generation kit: Abbott Diagnostics) using the chemiluminescence technique.

Results: In this study, in total, 249,461 units of blood were collected, of which the majority of donations were by male and replacement donors (RDs) comprising 230,757 (92.50%) and 188,047 (75.38%), respectively. The mean number of blood donations by replacement and male donors (MDs) was more than for voluntary donors (VDs) and female donors (FDs) (20894.11 ± 3041.71 RDs vs. 6823.77 ± 2332.96 VDs, p < 0.0001 and 25639.66 ± 2810.08 MDs vs. 2078.22 ± 828.16 FD, p < 0.0001), respectively. The overall prevalence of HCV was 0.63%, and all seropositive donors were male.

Conclusion: Replacement blood donation contributes to the major part of blood donation and is primarily done by males in this tribal population-dominant region of India. Seroprevalence of HCV is high in the population of this part of India, and there is a constant or slightly upward trend in hepatitis C infection among individuals.

## Introduction

Blood transfusion plays an essential role in saving the lives of many patients but concurrently bears the risk of passing on transfusion-transmitted infections (TTIs) from donor to recipient [1-3]. Blood and blood product transfusion service aims to provide ample, safe, low-risk, or risk-free blood and blood components to the needy. Each blood transfusion unit carries a 1% risk of noninfectious and infectious complications including TTIs [4]. Prevention of transfusion-associated infection during blood and blood components transfusion presents a significant challenge for transfusion medicine [5]. It has been made mandatory by the Drug and Cosmetic Act of 1945 to screen for major TTIs, namely, malaria, HIV-1 and 2, syphilis, hepatitis B, and hepatitis C, in all kinds of blood donations whether done by voluntary blood donors or replacement blood donors [6-7]. Transfusion medicine departments, along with screening all major TTIs, also provide an idea about the prevalence of transfusion-associated infections in healthy populations of communities [8]. Hepatitis C virus (HCV) transmission is associated with blood transfusion, perinatal exposure, contaminated needle prick injury, and sexual contact of carriers and family contacts [9]. Blood transfusion is one of the significant causes of transmission of HCV among individuals and is a major cause of post-transfusion hepatitis C infection [10].

In countries where blood is not screened routinely or not screened appropriately for major TTIs, the risk of getting HCV infection through transfusion is about 20-40% [11]. Another mode of transmission of HCV is needlestick injury, where a single prick carries a 5-15% risk of transmitting infection depending on the volume of inoculum [12]. Worldwide, around 50 million individuals are carrying chronic HCV infection; each year, around one million individuals get new HCV infections [13]. HCV causes a wide range of liver disorders, such as acute viral hepatitis, chronic viral hepatitis, liver cirrhosis, and hepatocellular carcinoma [14]. The World Health Organization aims to eliminate hepatitis C as a risk to community health by 2030 by reducing deaths and new infections [15]. This study is undertaken at a tertiary care center of a tribal population-dominant state of India to determine the seroprevalence of HCV in apparently healthy replacement and voluntary blood donors. As tribal populations are more prone to contracting HCV, this study is being done at our center.

## Materials and methods

Study design

This was a retrospective observational study done over nine years.

Study site and duration

This study was done in the Department of Transfusion Medicine and Blood Center of Rajendra Institute of Medical Sciences (RIMS), Ranchi, a tertiary care institute of Jharkhand state, a tribal population-dominant northern state of India where patients are referred from all over the state and neighboring states. The study was done over nine years from 2015 to 2023.

Study population and sampling

The study population comprised voluntary and replacement blood donors who donated blood at the Department of Transfusion Medicine and Blood Center during the study period.

Inclusion and exclusion criteria

Blood donors were selected as per the guidelines of the National Blood Transfusion Council, Ministry of Health and Family Welfare in October 2017. All blood donors were canvassed for medical history, examined clinically, and underwent laboratory tests. Persons aged 18-65 years, with body weight >45 kg, hemoglobin level >12.5 gm/dl, systolic blood pressure 100-140 mmHg, and diastolic blood pressure 60-90 mmHg and who had not donated blood in the preceding three months, were considered eligible for blood donation. Persons aged <18 years or >65 years; body weight <45 kg; hemoglobin level <12.5 gm/dl; suffering from cardiac disease, autoimmune disease, seizure disorders, bronchial asthma, renal failure, bleeding disorders, or hemoglobinopathies; and who had a history of high risk-behavior were not allowed for blood donation. Those who met the criteria for blood donation were included in the study, while the rest were excluded. Those who were included in the study were given a specific donor registration number and their informed consent was taken for the test of TTI, including an anti-HCV test in their blood sample.

Data collection

A questionnaire seeking information about personal details, demographic details, history of blood donation, medical history, and high-risk-behavior activities was used for collecting data from persons who gave consent and were found to be eligible for blood donation. Consent was also taken from them regarding testing for HCV and other TTIs on their blood sample. Data records of blood donors, including their hepatitis C and TTI status from January 1, 2015 to December 31, 2023, were retrieved from the Department of Transfusion Medicine and Blood Center and were recorded in prescribed proforma. Data of 249,461 blood donors were collected, of whom 188,047 were replacement donors, while 61,414 were voluntary donors.

Ethical approval and informed consent

Ethical approval was provided by the Institutional Ethics Committee of Rajendra Institute of Medical Sciences (RIMS), Ranchi, vide registration no. R 31/24 dated 10 May 2024. Informed consent was taken from each blood donor at the time of blood donation.

Method of hepatitis C detection

All voluntary and replacement blood donors were tested for anti-HCV antibodies in their blood sample through the chemiluminescence technique using a third-generation kit of Abbott Diagnostics (manufacturer: Abbott, model: Architect I 1000SR). Manufacturer guidelines were strictly followed. Donors, blood samples collected in ethylenediamine tetraacetic acid vials after blood donation were centrifuged at 10,000 rpm for 10 minutes, and their serums were separated in sample cups for testing of anti-HCV antibodies. All serum samples were recentrifuged, which showed results of >0.80 relative light units (RLUs). Blood samples that showed test results of 1.0 RLU or more and gray-zone results (0.90-0.99 RLU) were considered positive for anti-HCV antibodies. All positive donors having test results >1.0 RLU were counseled and referred to the Department of Medicine for further evaluation and treatment, whereas all donors with grey zone results (0.90-0.99 RLU) were counseled and advised to repeat testing after three months. All the reactive samples along with their corresponding blood units were discarded as per the standard operative procedure guidelines of the blood center. 

Statistical analysis

All collected data were stored in Microsoft Excel (Microsoft Corporation, USA) and were analyzed using Statistical Package for Social Sciences software (version 22.0. IBM Corp., Armonk, NY). Tables and graphs were made from available data. Percentages and proportions along with a 95% confidence interval were calculated for variables. A line graph was used to see the trend. The Wilson score interval test was used to estimate the proportion confidence interval, while the Student t-test was used to compare the means. A P value of <.05 was considered significant.

## Results

**Figure 1:**
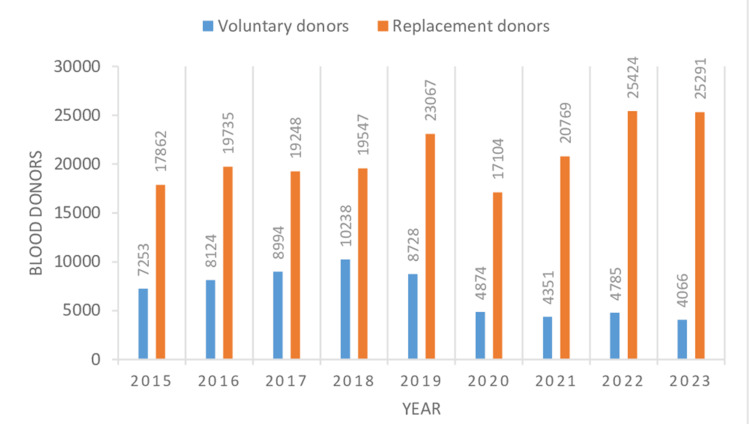
Year-wise distribution of voluntary and replacement blood donation.

**Figure 2:**
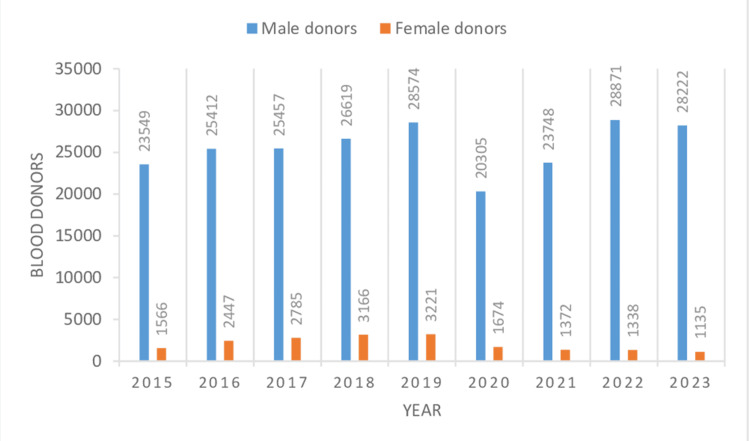
Year-wise distribution of male and female blood donors.

**Figure 3:**
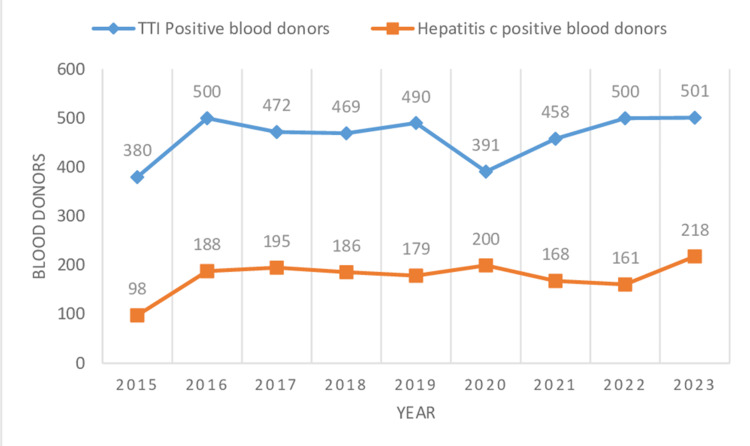
Year-wise trend of seroprevalence of total TTI and HCV TTI: transfusion-transmitted infection; HCV: hepatitis C virus

In the present study, the total number of blood units collected was 249,461 during the study from 2015 to 2023. Voluntary donors numbered 61,414 (24.61%), and 188,047 (75.38%) were replacement donors (Table 1, Figure 1). Male donors numbered 230,757 (92.50%), and 18,704 (7.49%) were female donors (Table 1, Figure 2). The highest number of blood donations was received in the year 2019, comprising 31,795 (12.74%) donations, whereas the lowest number of blood donations was received in the year 2020, comprising 21,979 (8.81%) donations, which was the year of the first wave of COVID in India. Voluntary donors donated the most units of blood in 2018 and the least number of units in 2023, consisting of 10,238 (16.67%) and 4,066 (6.62%) units, respectively. The most and least blood donations by replacement donors were done in 2022 and 2020, respectively, comprising 25,424 (13.52%) and 17,104 (9.09%) units of donation. Males and females donated the most blood in 2022 and 2019, comprising 28,871 (12.51%) and 3,221 (17.22%) units of donation, respectively, whereas they donated the least units of blood in 2020 and 2023, which accounted for 20,305 (8.79%) and 1,135 (6.06%) units of blood donation, respectively. There was a significant difference in the mean numbers of blood donations by replacement and voluntary blood donors per year, which were 20,894.11 ± 3,041.71 and 6,823.77 ± 2,332.96 (p < 0.0001), respectively, suggesting replacement donations as the main form of blood donation (Table 1). The mean numbers of male and female blood donors per year were 25,639.66 ± 2,810.08 and 2,078.22 ± 828.16 (p < 0.0001), respectively, which gives an idea that male blood donors are the primary blood donors (Table 1). On screening of 249,461 blood donors, 4,161 (1.66%) donors showed TTI positivity for (HIV1, HIV2, HBV, HCV, syphilis, and malaria and 1,593 (0.63%) donors were positive for HCV (Table 2, Figure 3). All seropositive donors of HCV were male (Table 3). Trends of seroprevalence of hepatitis C in blood donors showed an increase in hepatitis C positivity from the year 2015 to 2016, whereas after 2016, there was a plateau in hepatitis C positivity among blood donors up to 2022, and again, there was an increase in positivity from 2022 to 2023.

**Table 1:** Year- and gender-wise distribution of blood donors VD: voluntary donors; RD: replacement donors; n: numbers; %: percentage Data are presented as n and n (%). A p-value of <.05 is considered significant. The mean and standard deviation of replacement donors, voluntary donors, male donors, and female donors are obtained from values in Table 1.

	Sl. no.	Year	Total units of blood collection (VD+RD) (n)	Total units of blood collection by voluntary donations (n)	Total units of blood collection by replacement donations (n)	Total male donors (VD+RD) (n)	Total female donors (VD+RD) (n)
	1	2015	25,115	7,253	17,862	23,549	1,566
	2	2016	27,859	8,124	19,735	25,412	2,447
	3	2017	28,242	8,994	19,248	25,457	2,785
	4	2018	29,785	10,238	19,547	26,619	3,166
	5	2019	31,795	8,728	23,067	28,574	3,221
	6	2020	21,979	4,875	17,104	20,305	1,674
	7	2021	25,120	4,351	20,769	23,748	1,372
	8	2022	30,209	4,785	25,424	28,871	1,338
	9	2023	29,357	4,066	25,291	28,222	1,135
		Total n(%)	249,461 (100%)	61,414 (24.61%)	188,047 (75.38%)	230,757 (92.50%)	18,704 (7.49%)

**Table 2:** Year-wise prevalence of total TTIs (HBV, HCV, HIV1, HIV2, malaria, and syphilis) and HCV among blood donors. Data are presented as numbers (n) and proportion with 95% confidence interval (CI). The Wilson score interval test is used to estimate the proportion confidence interval. TTI: transfusion-transmitted infections; CI: confidence interval; HCV: hepatitis C virus

Sl. No.	Year	Total collection (n)	Total TTI-positive donors (n)	Proportion with 95% CI of total TTI-positive donors	Total HCV-positive donors (n)	Proportion with 95% CI of total HCV-positive donors
1	2015	25,115	380	1.51 (1.36-1.66)	98	0.39 (0.31-0.46)
2	2016	27,859	500	1.79 (1.63-1.95)	188	0.67 (0.57-0.77)
3	2017	28,242	472	1.67 (1.52-1.82)	195	0.69 (0.59-0.78)
4	2018	29,785	469	1.57 (1.43-1.71)	186	0.62 (0.54-0.72)
5	2019	31,795	490	1.54 (1.40-1.67)	179	0.56 (0.48-0.64)
6	2020	21,979	391	1.77 (1.60-1.95)	200	0.90 (0.78-1.03)
7	2021	25,120	458	1.82 (1.65-1.98)	168	0.66 (0.56-0.76)
8	2022	30,209	500	1.65 (1.51-1.79	161	0.53 (0.45-0.61)
9	2023	29,357	501	1.70 (1.55-1.85)	218	0.74 (0.64-0.84)
Total		249,461	4161	1.66 (1.61-1.71)	1593	0.63 (0.60-0.66)

**Table 3:** Year- and gender-wise distribution of HCV-positive blood donors Data are presented in numbers (n) and proportion with 95% confidence interval (CI). The Wilson score interval test is used to estimate the proportion confidence interval TTI: transfusion-transmitted infection; CI: confidence interval, HCV: hepatitis C virus

Year	Total TTI-positive donors (n)	Hepatitis C-positive male donors (n)	Proportion with 95% CI of hepatitis C-positive male donors	Hepatitis C-positive female donors (n)	Proportion with 95% CI of hepatitis C-positive female donors
2015	380	98	25.78 (21.39-30.19)	00	0.00
2016	500	188	37.6 (33.35-41.85)	00	0.00
2017	472	195	41.31 (36.87-45.76)	00	0.00
2018	469	186	39.65 (35.23-44.09)	00	0.00
2019	490	179	36.53 (32.27-40.79)	00	0.00
2020	391	200	51.15 (46.2-56.11)	00	0.00
2021	458	168	36.68 (32.27-41.09)	00	0.00
2022	500	161	32.2 (28.1-36.3)	00	0.00
2023	501	218	43.51 (39.17-47.85)	00	0.00
Total	4,161	1,593	38.28 (36.81-39.76)	00	0.00

## Discussion

Among the many services of healthcare delivery, transfusion medicine services constitute an essential and crucial part of its proper functioning. The main purpose of transfusion medicine services is to provide a safe, ample, accessible, and efficient supply of blood and blood-related components at different levels [16]. Over the years, in developed countries, there has been a highly significant reduction in the risk of TTIs related to blood and blood product transfusion [17]. Conversely, in developing countries, the risk of TTIs due to blood transfusion-related services remains significant compared to developed countries. India, through its national blood policy, has taken stringent action to reduce TTI and provide a safe and adequate blood supply. The cornerstone of this strategy is collecting blood donations only from nonremunerated replacement and voluntary blood donors, refusing all paid donations, a thorough screening of all major TTI, and avoiding blood transfusion in cases where it is not necessary [18]. The present study showed a higher number of replacement donors (75.38%) than voluntary donors (24.61%), which was comparable with other studies in other regions of India, which showed the predominance of replacement donors, by Singh et al. (82.4%) [19], Kakkar et al. (94.7%) [20], Singh et al. (84.43) [21], Pahuja et al. (99.48%) [22], Arora et al. (68.6%) [23], Mondal et al, (96.3%) [24], Pawan et al. (84.87%) [25], Garg et al. (90.1) [26], and Pokhrel et al. (80.3%) [27], whereas the present study showed a contrast with the findings of the predominance of voluntary donors by Patel et al. (95.56%) [28], Kaur et al. (65.86%) [29], Pallavi et al. (64.78%) [30], Dhariwal et al. (80.4%) [31], and Ryhan (69.62%) [32]. A lower number of voluntary donors at our center and in most parts of India is due to multiple reasons prevalent in our society. The lack of motivation for donating blood, a smaller number of voluntary blood donation camps, misbeliefs and misconceptions about blood donation, fear of blood donation, and time required for blood donation are some of the factors that result in fewer people turning out for voluntary blood donation. Some other factors, such as lack of information about blood donation, poor attitude of blood bank staff, fear of misuse of blood, fear of needles or pins, fear of contagion, fear of weakness, dizziness after blood donation, and absence of rewards or gifts contribute to the reduced number of voluntary blood donors. Voluntary blood donation is considered the safest form of blood donation because the number of TTIs is much smaller in comparison to replacement donation. The present study showed predominance of male donors, which was comparable with other studies by Mondal et al. (98.3%) [24], Kaur et al. (98.17%) [29], Ryhan et al. (98.45%) [32], Pawan et al. (96.12%) [25], Dhariwal et al. (97.5%) [31], Pahuja et al. (97.24%) [22], Saini et al. (99.7%) [33], and Shah et al. (99.08%) [34]. Reduced participation of female blood donors in comparison to male blood donors in our study can be explained by the fact that female education is given much lower priority in India in comparison to male education, and these less educated females know very little about blood donation and are also less motivated for blood donation. Other factors, such as large numbers of females being anemic, low body weight, fear of the process of blood donation, more adverse effects in females like weakness, and dizziness after blood donation in comparison to males, previous deferral from blood donation, and also menstruation, pregnancy, and breastfeeding are responsible for the avoidance of blood donation by females. Less blood donation by females also leads to fewer instances of voluntary blood donation and in turn reduced supply of safe blood.

In the present study, the seroprevalence of HCV was found to be 0.63% (Table 2). As compared to our study, the seroprevalence of HCV was reported in blood donors in other parts of India by Garg et al. (0.28%) from western India [26], Mandal et al. (0.62%) from Darjeeling [35], Jindal et al. (1.76%) from north India [36], Saini et al. (0.07%) from Indore [33], Mukherjee et al. (0.044%) from western Odisha [37], Shaiji et al. (0.72%) from Trivandarum [38], Meena et al. (0.57%) from Delhi [39], Pahuja et al. (0.66%) from Delhi [22], Kaur et al. (2.44%) from North India [29 ], Afrose et al. (0.22%) from Aligarh [40], Ryhan et al. (0.22%) from Srinagar [32], Shah et al. (0.33%) from Ahmedabad [34], Gupta et al. (1.45%) from Ludhiana [41], Bagde et al. (0.065%) from Rajnandgaon, Chhattisgarh [42], Pawan et al. (0.50%) from Delhi [25], Sawke et al. (0.57%) from Bhopal [43], Patel et al. (0.14%) from Gujarat India [44], and Dhariwal et al. (0.71%) from Rajasthan [31]. However, reported seroprevalence of HCV in countries other than India was given by Abebe et al. (0.64%) from Western Oromia, Ethiopia [45], Mobarki et al. (1.09%) from Jazan region of Saudi Arabia [46], Ahmed et al. (1.75%) from Karachi, Pakistan [47], and Alharazi et al. (2.0%) from Sana’a, Yemen [48]. The higher prevalence of hepatitis C in apparently healthy blood donors in our study can be explained by the fact that persists in abundance in our society. Unqualified doctors in India practice unsafe therapeutic practices, such as not sterilizing instruments, multiple uses of the same needles, blood banks not following guidelines for testing of TTIs, intravenous drug use, accidental exposure of healthcare workers, dental procedures, non-use of condoms by sex workers, large numbers of migrant population, tattooing, body piercing, and shaving by village barbers are factors responsible for hepatitis C infection increasing its prevalence. A few other factors, such as multiple blood transfusions in patients with hemoglobinopathies, clotting disorder, renal failure cross-contamination from dialysis circuits, and unsafe blood transfusion in trauma, medical, and surgical patients, also lead to HCV infection, causing a rise in its prevalence. 

Similar to the present study, there were no HCV-reactive female blood donors [29]. This difference might be due to the lesser participation of female donors in the present study. Males are more prone to harboring the risk of infection [49]. The current study showed either a plateau or an increase in hepatitis C positivity in blood donors, which is similar to the results of Saini et al., who found an increase in seroprevalence of hepatitis C in blood donors over the years [33]. The constant or upward trend in the prevalence of hepatitis C infection in the present study can be explained by the fact that risk factors for transmission of HCV are not controlled adequately. The National Virus Hepatitis Control Program was started in India in 2018 to reduce the incidence and deaths due to different hepatitis viruses for which strict measures have to be taken.

India is a country with the largest population in the world. Its huge population contributes to large numbers of patients with medical and surgical illnesses and also patients with trauma and road traffic accidents. For the management of these patients, safe blood and blood components in large numbers are required. Voluntary blood donation is considered the safest form of blood donation because it is associated with the least TTIs. The World Health Organization (WHO) aspires for 100% of blood donation by voluntary blood donors. Thus, to increase the number of blood donations and to maintain its safety, voluntary blood donation should be encouraged. Education of people; encouragement by motivators for blood donation; organization of large numbers of voluntary blood donation camps; promotional activities through television, radio, and posters; increased participation of females in blood donation; and strict regulatory measures will lead to an increase in blood donation, which will be primarily voluntary and safe.

Limitation

This study was a single-center study, so a generalization of results cannot be made. Because it is a retrospective observational study, a few variables may be missing from the collected data. Detection of HCV was done by an antibody-based test, so a few people could have evaded detection who were in the window period at the time of blood donation. One more limitation was the failure to detect the genotype of HCV because genotyping was not done due to the lack of availability of the required test at our institute.

## Conclusions

Our study showed a high seroprevalence of HCV among blood donors in the tribal-preponderant region of India. Participation of RDs and MDs was more than VDs and FDs. All HCV-reactive donors were male. Constant upgradation of public awareness, knowledge, and perception about blood donation will lead to increased participation of FDs with the implementation of stringent donor selection, and sensitive screening tests will increase blood safety and decrease the incidence of transmission of HCV and other TTIs. Seroprevalence of hepatitis C infection has reflected a constant or upward trend over the years, for which necessary action should be taken to meet the goal of the WHO to eliminate hepatitis C as a public health threat by 2030.
